# Antihypertensive Interventions in Acute Ischemic Stroke: A Systematic Review and Meta-Analysis Evaluating Clinical Outcomes Through an Emergency Medicine Paradigm

**DOI:** 10.7759/cureus.47729

**Published:** 2023-10-26

**Authors:** Hany A Zaki, Stuart A Lloyd, Amr Elmoheen, Khalid Bashir, Wael Abdelrehem Elnabawy Elsayed, Mohammed Gafar Abdelrahim, Kaleem Basharat, Aftab Azad

**Affiliations:** 1 Emergency Medicine, Hamad Medical Corporation, Doha, QAT; 2 Emergency Medicine, Hamad General Hospital, Doha, QAT; 3 Medicine, Qatar University, Doha, QAT

**Keywords:** risk ratio, meta-analysis, a systematic review, : ischemic stroke, stroke, hypertension

## Abstract

High blood pressure (HBP) is usually prominent after the onset of acute ischemic stroke (AIS). Although previous studies have found that about half of patients with AIS have a background of hypertension, there is no clear etiology for HBP in AIS. The literature reveals discrepancies in the relationship between HBP and clinical outcomes of AIS, pointing toward the contested effect of blood pressure (BP) reduction clinical outcomes. Thus, the potential benefits and hazards of HBP treatment were explored in the context of clinical outcomes after AIS.

An electronic database and a manual search were carried out to identify all the articles related to this topic and published between 2000 and January 2023. The Review Manager software was also used to perform the meta-analysis and quality appraisal. In analyses related to patients not treated with reperfusion therapies, mortality, and dependency outcomes were categorized as short-term (<3 months) or long-term (≥3 months).

Our search strategy yielded 2459 articles, of which only 15 met the inclusion criteria. The results of our meta-analysis demonstrate that in patients not treated with reperfusion therapies, BP lowering had no significant impact on either short-term or long-term mortality (risk ratio (RR): 1.18; 95% confidence interval (CI): 0.81-1.73; p = 0.39, and RR: 1.04; 95% CI: 0.77-1.40; p = 0.81, respectively) and dependency (RR: 1.12; 95% CI: 0.97-1.30; p = 0.11, and RR: 0.98; 95% CI: 0.90-1.07; p = 0.61, respectively). Furthermore, BP lowering prior to reperfusion showed no significant effect on mortality (RR: 0.7; 95% CI: 0.23-2.26; p = 0.58), but it did significantly reduce the risk of dependency (RR: 0.89; 95% CI: 0.85-0.94; p < 0.00001). When the dataset was restricted to patients who had successful reperfusion, intensive BP lowering (target systolic BP <120 mmHg) was found to increase the risk of dependency (RR: 1.23; 95% CI: 1.09-1.39; p = 0.0009). In addition, BP reduction had an insignificant effect on the risk of recurrent strokes and combined vascular events (RR: 1.00; 95% CI: 0.54-1.84; p = 1.00, and RR: 0.99; 95% CI: 0.70-1.41; p = 0.95, respectively).

Lowering BP in patients not treated with reperfusion therapies is not beneficial in reducing the risk of either short or long-term mortality and dependency. However, BPR before reperfusion reduces the risk of dependency, while aggressive BPR (target systolic blood pressure (SBP) <120 mmHg) after successful reperfusion increases the risk of dependency. Therefore, we recommend BPR as early as possible for patients undergoing reperfusion therapies but suggest against aggressive BPR in patients who have undergone successful reperfusion.

## Introduction and background

High blood pressure (HBP) is a common condition observed in patients with acute ischemic stroke (AIS). The World Health Organization has reported that HBP affects up to 80% of AIS patients [1,2]. Conversely, low or low-normal blood pressure (BP), which is less frequent, affects about 4% of these patients [2]. However, it is worth noting that the BP usually returns to normal in about two-thirds of the patients within the first week [3]. The cause of HBP in AIS is poorly understood, but one research paper shows that over half of the patients typically have a history of hypertension [4]. Other potential explanations for HBP in AIS patients include the stress of being hospitalized, increased cardiac output, neuro-endocrine system activation, and complications from the stroke, such as urinary retention and increased intracranial hypertension. Additionally, it is suggested that after an acute stroke, cardiac baroreceptor sensitivity may be impaired, which could explain HBP [5].

Previous studies have shown some contradictory results on the relationship between HBP and outcomes of AIS. A large trial of 17,398 patients with ischemic stroke reported that HBP was associated with poor outcomes [6]. The trial results showed that for patients with systolic BP (SBP) above 150 mmHg, an increase of 10 mmHg was associated with a 3.8% increase in early deaths and an insignificant increase (1.1%) in six-month death or dependency. The study also showed that recurrent ischemic stroke was independently associated with an increase in BP. The data showed that the frequency of early recurrence was 4.2% for every 10 mmHg rise in SBP. In Contrast, Jorgensen et al. [7] reported that HBP appeared to reduce the risk of developing ischemic stroke progression. This contradictory information suggests that managing HBP may have an uncertain impact on the clinical outcomes of AIS. The aim of this study was to evaluate the clinical effects and risks of managing HBP in AIS patients.

## Review

Methodology

Protocol and Registration

This systematic review was designed according to the Cochrane Collaboration guidelines, and results were reported as per the Preferred Reporting Items for Systematic Review and Meta-Analyses guidelines [8]. It was registered on the International Prospective Register of Systematic Reviews (CRD42023394581). All authors have reviewed the article.

Data Sources and Literature Search

Published observational studies and randomized trials that reported HBP control in AIS were sought from five electronic databases, including EMBASE, Cochrane Library, MEDLINE, ScienceDirect, and Google Scholar. In addition to the database search, the bibliographies of the selected articles were reviewed for further reference. Supplementary searching was performed using snowballing and hand searching of key medical journals. The literature search was performed independently by two research members in November 2022. A repeat search was performed in January 2023. The search strategy used to obtain studies from these electronic databases employed various keywords and the Boolean operators “AND” and “OR” (see Appendix).

Eligibility Criteria

Two reviewers were assigned to assess articles for inclusion and exclusion. The search was restricted to studies on humans and published in English. Studies that recruited pediatric or adult patients with HBP after an AIS were included. The exclusion criteria were studies that included less than 10 patients (since statistical power depends on the sample size); studies that examined the correlation between HBP and the clinical results of AIS; letters to the editor, systematic reviews and meta-analyses, and case reports; studies that included only patients with low or low-normal BP after AIS; and studies that included patients with acute stroke but did not distinguish the data for patients with ischemic stroke.

Outcome Measures

The primary outcomes of the present study were mortality and dependency, while the secondary outcome was serious adverse events. Dependency was defined as a score of 3 to 6 on the modified Rankin scale (mRS) [9].

Although mortality and dependency were considered the main outcomes, our primary aim was to determine whether lowering BP in AIS patients subjected or not subjected to reperfusion therapies would improve the outcomes. Outcomes for patients undergoing reperfusion therapies were further grouped based on the timing of BPR (i.e., before or after successful reperfusion). When it was applicable, subgroup analysis based on the follow-up period was performed (i.e., short (<3 months) or long-term (≥3 months)).

In addition, we investigated the impact of factors such as drug class and treatment timing after symptom onset on mortality and dependency outcomes.

Data Extraction and Quality Assessment

Two reviewers extracted data from the selected studies into a standardized form (Table 1).

**Table 1:** Characteristics of included studies RCT: randomized controlled trial, TIA: transient ischemic attack, NA: not available, SBP: systolic blood pressure, BP: blood pressure, NR: not required, SAE: serious adverse events, M/F: male/female.

Study	Study design	Country	Participant characteristics				BPR intervention and time to treatment	Target BP	Timing of reperfusion therapy	Outcomes
			Sample (n)	M/F	HBP definition	Comorbidities (n)				
He et al., 2014 [10]	RCT	China	4071	2604/1467	SBP between 140 mmHg and less than 220 mmHg	Hypertension (1610), hyperlipidemia (137), diabetes (369), coronary heart disease (216)	Antihypertensive medications administered 24 hours after stroke onset	SBP <140 or HBP <90 mmHg within 7 days	NA	Mortality, dependency, and SAE
Potter et al., 2009 [11]	RCT	United Kingdom	99	NR	SBP >160 mmHg	NR	Lisinopril and intravenous labetalol administered 24 hours after the stroke onset	SBP: 145–155 mmHg within 8 hours	NA	Dependency
Ahmed et al., 2000 [12]	RCT	United Kingdom	295	NR	NR	NR	Low-dose and High-dose intravenous nimodipine administered 24 hours after stroke onset	NR	NA	Mortality and dependency
Bath et al., 2009 [13]	PRoFESS trial subgroup analysis	United Kingdom	1360	884/476	SBP ≥140 mmHg or DBP ≥90 mmHg	Previous stroke/TIA (344), atrial fibrillation (24), hypertension (956), diabetes (374), hyperlipidemia (547), left ventricular hypertrophy (159), ischemic heart disease (199)	Telmisartan administered 72 hours after the stroke onset	SBP <140 or DBP <90 mmHg	NA	Mortality, dependency, and SAE
Eveson et al., 2007 [14]	RCT	United Kingdom	40	25/15	SBP ≥140 mmHg or DBP ≥90 mmHg	Previous stroke/TIA (6), atrial fibrillation (7), hypertension (27), diabetes (9)	Lisinopril administered within 24 hours after stroke onset	SBP <140 or HBP <90 mmHg within 7 days	NA	Mortality and dependency
Martin-Schild et al., 2008 [15]	Retrospective observational study	United States	178	115/167	SBP ≥185 mmHg or DBP ≥110 mmHg	NR	Labetalol administered within 3 hours after stroke onset	SBP <185 or HBP <110 mmHg	3 hours after stroke onset	Mortality and dependency
Oh et al., 2015 [16]	RCT	South Korea	393	232/161	SBP: 150–185 mmHg	Previous stroke/TIA (56), atrial fibrillation (35), hypertension (292), diabetes (128), dyslipidemia (112)	Valsartan administered within 24 and 48 hours of stroke onset	15% SBP reduction from baseline or to 145 mmHg within 1 day	NA	Mortality, dependency, and SAE
Berge et al., 2015 [17]	RCT	Norway	3028	1567/1461	NR	NR	Blood pressure lowering treatment in the first 24 hours of stroke onset	NR	6 hours after stroke onset	Mortality and dependency
Horn et al., 2001 [18]	RCT	Netherlands	261	NR	NR	Previous stroke (24), cardiac disease (106)	Nimodipine administered 6 hours after stroke onset	NR	NA	Dependency
Xu et al., 2017 [19]	CATIS trial subgroup analysis	China	4071	2604/1467	SBP between 140 mmHg and less than 220 mmHg	Hypertension (1610), hyperlipidemia (137), diabetes (369), coronary heart disease (216)	Antihypertensive medications administered 24 hours after stroke onset	SBP <140 or HBP <90 mmHg within 7 days or 10–25% SBP reduction within 24 hours	NA	Mortality and dependency
Yuan et al., 2021 [20]	RCT	China	483	269/214	SBP: 150–210 mmHg	Ischemic stroke (87), hemorrhagic stroke (40), coronary artery disease (138), renal disease (10), diabetes (97), hypertension (389)	Antihypertensive treatment administered 72 hours after stroke onset	10–15% SBP reduction within 24 hours and maintained for 1 week	NA	Mortality and dependency
Kaste et al., 1994 [21]	RCT	Finland	350	235/115	NR	Hypertension (141), coronary heart disease (121), diabetes (59)	120 mg of nimodipine administered 48 hours after stroke onset	NR	NA	Mortality and dependency
Schrader et al., 2003 [22]	RCT	Germany	339	102/237	SBP ≥200 mmHg or DBP ≥110 mmHg 6 to 24 hours after admission or SBP ≥180 mmHg or DBP ≥105 mmHg 24 to 36 hours after admission	Coronary heart disease (41), diabetes (74), hyperlipidemia (88)	4 mg candesartan cilexetil administered 24 hours after stroke onset	SBP <140 or HBP <90 mmHg (office blood pressure) or SBP <135 or HBP <85 mmHg (mean daytime blood pressure, automatic blood pressure monitoring)	NA	
Mazighi et al., 2021 [23]	RCT	France	318	153/165	NR	Hypertension (223), diabetes (67), hypercholesterolemia (114), previous stroke or TIA (46)	Antihypertensive drugs administered 24 hours after reperfusion therapy	SBP: 100–129 mmHg within 1 hour or SBP: 130–185 mmHg within 1 hour	285 minutes after stroke onset for intensive BPR group and 297 minutes after stroke onset for standard BPR group	Mortality and dependency
Yang et al., 2022 [24]	RCT	China	816	506/310	SBP ≥ 140 mmHg for >10 minutes after successful reperfusion	Hypertension (528), previous stroke (246), coronary artery disease (110), valvular heart disease (33), other heart disease (35), atrial fibrillation (182), diabetes (163), hypercholesterolaemia (27)	Antihypertensive drugs administered 24 hours after endovascular thrombectomy	SBP <120 mmHg or SBP: 140–180 mmHg within 1 hour and maintained for 72 hours	<6 hours or ≥6 hours after stroke onset	Mortality and dependency

**Figure 1:**
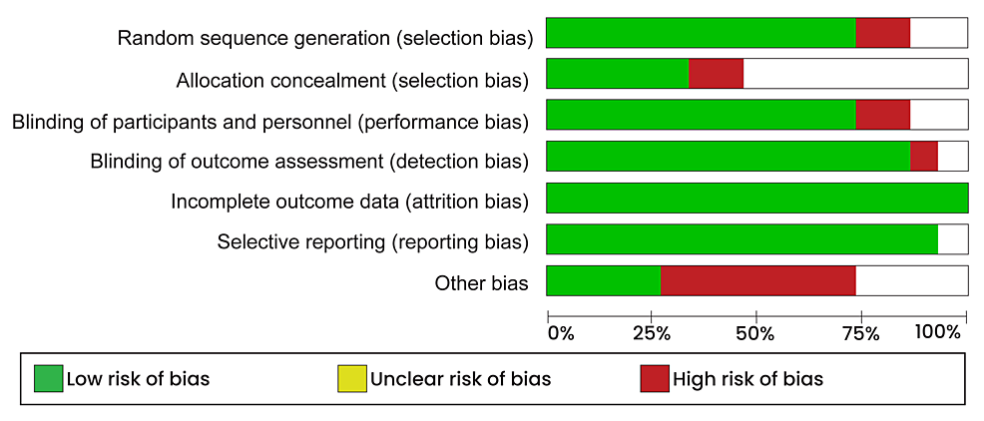
Risk of bias graph

**Figure 2:**
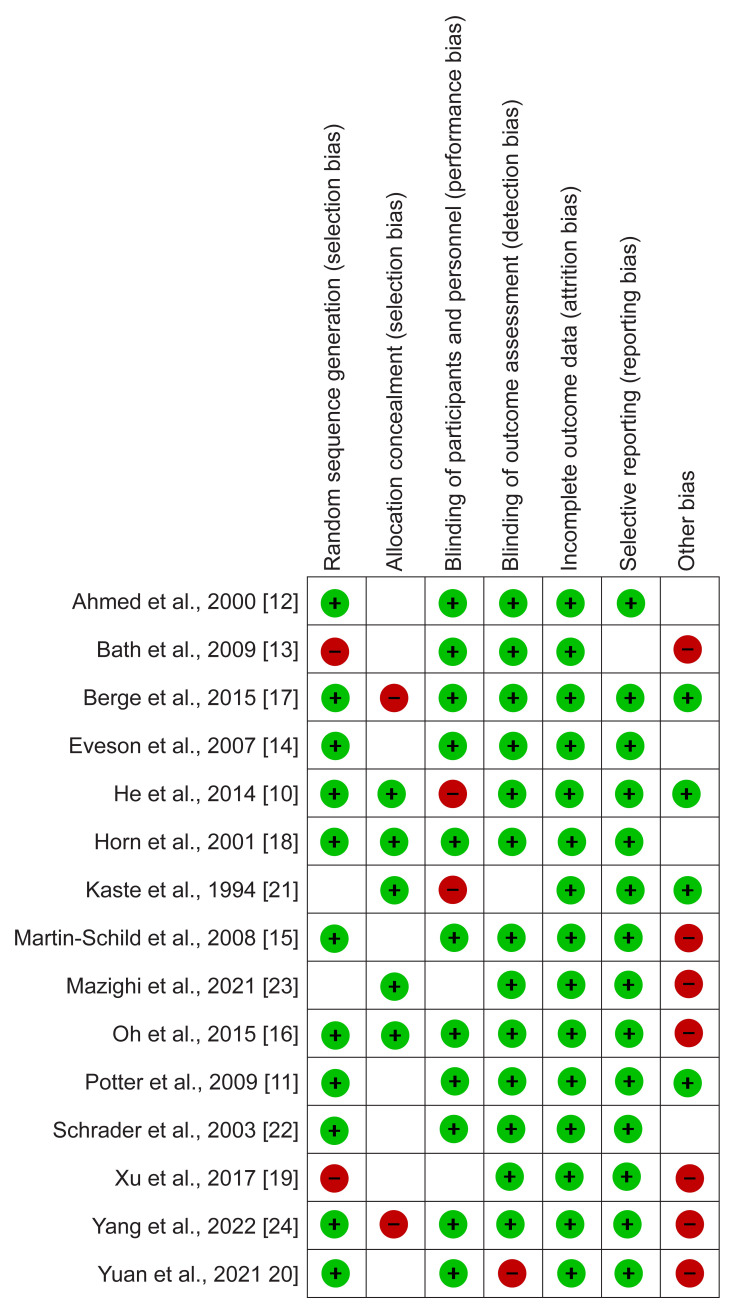
Risk of bias summary Data from He et al., 2014 [10], Potter et al., 2009 [11], Ahmed et al., 2000 [12], Bath et al., 2009 [13], Eveson et al., 2007 [14], Martin-Schild et al., 2008 [15], Oh et al., 2015 [16], Berge et al., 2015 [17], Horn et al., 2001 [18], Xu et al., 2017 [19], Yuan et al., 2021 [20], Kaste et al., 1994 [21], Schrader et al., 2003 [22], Mazighi et al., 2021 [23], Yang et al., 2022 [24]

When there was a discrepancy in extracted data, a third reviewer was tasked to reconcile the differences between the two reviewers. The quality assessment of evidence from all included studies was performed using the risk of bias tool within the Review Manager software (RevMan 5.4.1). The assessment was conducted independently, categorizing various study elements into selection, performance, attrition, and reporting bias. The quality assessment results are shown in Figure 1, which presents the risk of bias graph, and Figure 2, which presents the risk of bias summary.

Data Synthesis

The Review Manager software was used to calculate the overall impact of hypertension control on the clinical outcomes of AIS. All outcomes analyzed in the current study were binary; therefore, the pooled effect size was calculated using the risk ratio (RR). We also quantified the heterogeneity between studies using the I2 statistics; any value of I2>50% indicated significant heterogeneity. A p-value of <0.05 indicated a statistical difference after employing a confidence interval of 95% for the analysis. Final outcomes of our meta-analysis were then illustrated in the form of forest plots.

Results

Study Selection

**Figure 3:**
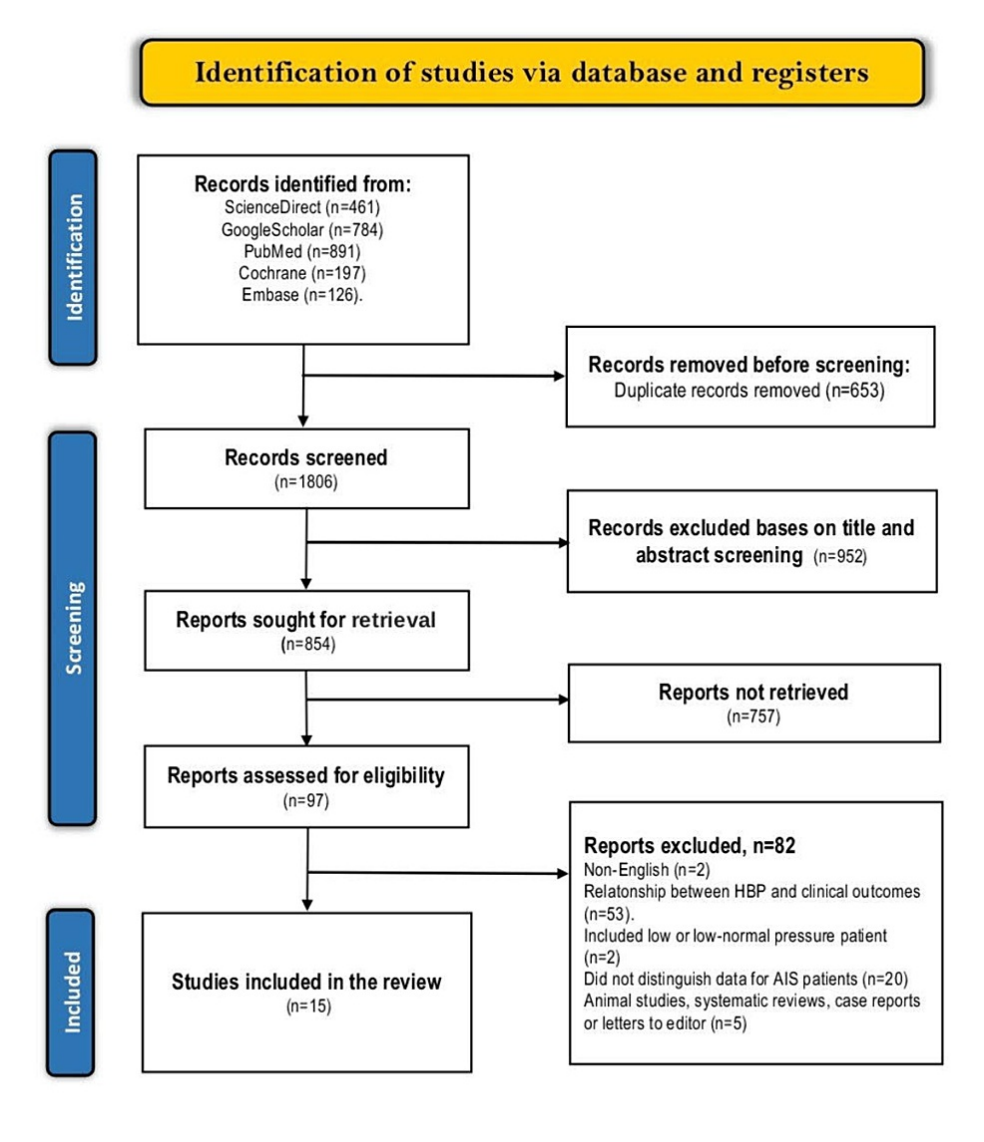
PRISMA flow diagram of the literature search results PRISMA: Preferred Reporting Items for Systematic Reviews and Meta-Analyses, HBP: high blood pressure, AIS: acute ischemic stroke

Our search criteria resulted in 2459 articles, of which 1605 were excluded based on the duplicate check and screening criteria. Of the 854 remaining articles, 757 were not retrieved because they were either ongoing trials or the full articles were not available, and the rest were evaluated based on the eligibility criteria. And 15 of the 97 remaining articles fulfilled the inclusion criteria, while the rest were excluded. The full selection criterion is summarized in the PRISMA flow chart (Figure 3).

Mortality

**Figure 4:**
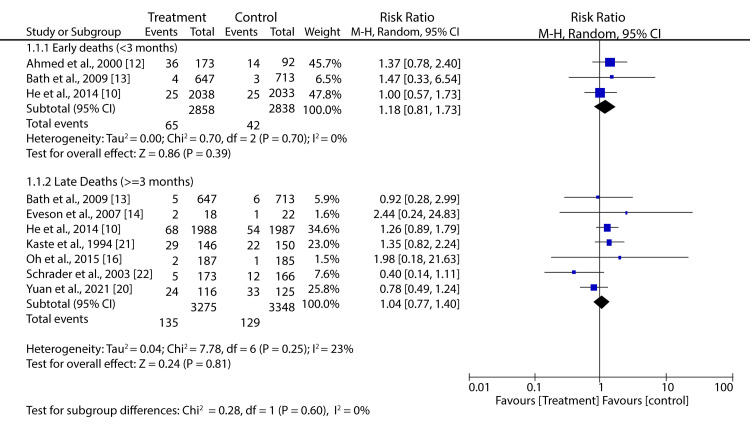
Forest plot showing the effect of HBP control on mortality for AIS patients not undergoing reperfusion treatment He et al., 2014 [10], Ahmed et al., 2000 [12], Bath et al., 2009 [13], Eveson et al., 2007 [14], Oh et al., 2015 [16], Yuan et al., 2021 [20], Kaste et al., 1994 [21], Schrader et al., 2003 [22]

Pooling data from eight trials with a random-effect model showed that BPR in patients not treated with reperfusion therapies did not affect the risk of short-term or long-term mortality (RR: 1.18; 95% CI: 0.81-1.73; p = 0.39, and RR: 1.04; 95% CI: 0.77-1.40; p = 0.81, respectively) (Figure 4).

**Figure 5:**
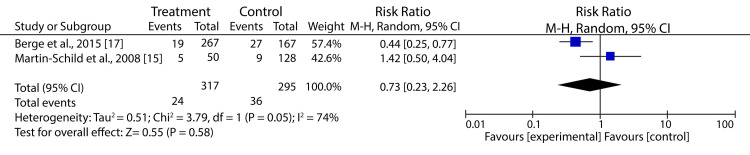
Forest plot showing the effect of HBP control prior to reperfusion treatment on Mortality of AIS patients Martin-Schild et al., 2008 [15], Berge et al., 2015 [17]

Similarly, BPR before reperfusion therapies had no significant effect on mortality (RR: 0.73; 95% CI: 0.23-2.26; p = 0.58) (Figure 5).

**Figure 6:**
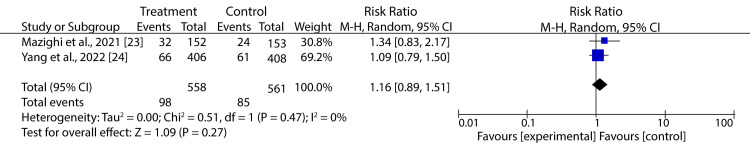
Forest plot showing the effect of HBP control after successful reperfusion treatment on mortality of AIS patients Mazighi et al., 2021 [23], Yang et al., 2022 [24] AIS: acute ischemic stroke, HBP: high blood pressure

Furthermore, our meta-analysis on the impact of BPR after successful reperfusion therapies revealed no considerable difference in mortality between the treatment and control groups (RR: 1.16; 95% CI: 0.89-1.51; p = 0.27) (Figure 6).

In addition, our pooled analysis suggests that the class of drug and time to treatment does not influence the mortality outcomes (Table 2).

**Table 2:** Meta-analysis results on the effect of class of drug and timing of treatment on mortality and dependency CI: confidence interval, N/A: not available

Factor	Outcome	Subgroup	Study analyzed	RR (95% CI)	p-Value	I^2 ^(%)
Class of drug	Mortality	Calcium channel blockers	2	1.94 (0.93–4.03)	0.08	72
		Angiotensin II receptor antagonists	3	0.77 (0.34–1.75)	0.53	28
		ACE inhibitors	1	2.44 (0.24–24.83)	0.45	NA
		Beta-blockers	1	1.42 (0.50–4.04)	0.51	NA
	Dependency	Calcium channel blockers	3	1.18 (0.99–1.41)	0.06	34
		Angiotensin II receptor antagonists	2	1.12 (0.91–1.37)	0.29	0
		ACE inhibitors	2	1.23 (0.90–1.69)	0.20	0
Time to treatment	Mortality	<24 hours	2	1.38 (0.96–1.99)	0.08	0
		≥24 hours	8	0.95 (0.64–1.41)	0.80	58
		≥72 hours	2	0.84 (0.56–1.27)	0.41	0
	Dependency	<24 hours	3	1.10 (0.98–1.23)	0.10	0
		≥24 hours	8	1.00 (0.88–1.14)	0.98	77
		≥72 hours	2	0.94 (0.83–1.06)	0.28	68

Dependency

**Figure 7:**
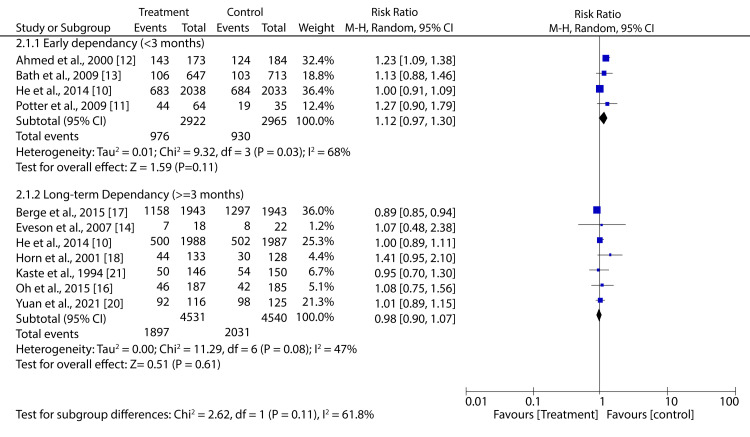
Forest plot showing the effect of HBP control on dependency of AIS patients not undergoing reperfusion treatment He et al., 2014 [10], Potter et al., 2009 [11], Ahmed et al., 2000 [12], Bath et al., 2009 [13], Eveson et al., 2007 [14], Oh et al., 2015 [16], Berge et al., 2015 [17], Horn et al., 2001 [18], Yuan et al., 2021 [20], Kaste et al., 1994 [21] AIS: acute ischemic stroke, HBP: high blood pressure

When we pooled data for patients not treated with reperfusion therapies, we found that there was no considerable difference between patients receiving antihypertensive medications and control on short- and long-term dependency (RR: 1.12; 95% CI: 0.97-1.30; p = 0.11 and RR: 0.98; 95% CI: 0.90-1.07; p = 0.61, respectively) (Figure 7).

**Figure 8:**
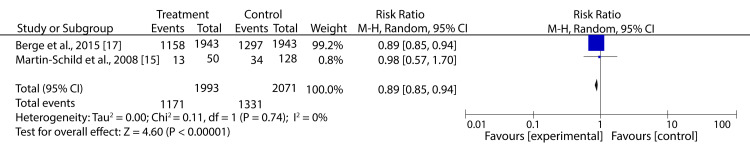
Forest plot showing the effect of HBP control prior to reperfusion treatment on the dependency of AIS patients Martin-Schild et al., 2008 [15], Berge et al., 2015 [17]

However, data pooled from two trials with AIS patients revealed that BPR before reperfusion treatments significantly reduced the risk of dependency compared to control (RR: 0.89; 95% CI: 0.85-0.94; p < 0.00001) (Figure 8).

**Figure 9:**
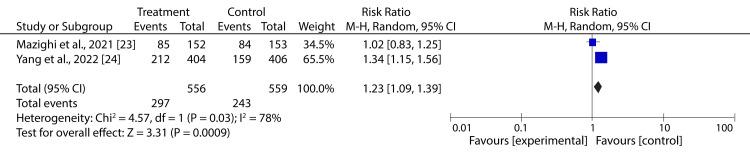
Forest plot showing the effect of HBP control after successful reperfusion treatment on dependency of AIS patients Mazighi et al., 2021 [23], Yang et al., 2022 [24] AIS: acute ischemic stroke, HBP: high blood pressure

Conversely, our meta-analysis suggests that aggressive BPR (target SBP <120 mmHg) after successful reperfusion treatment significantly increases the risk of dependency compared to control (RR: 1.23; 95% CI: 1.09-1.39; p = 0.0009) (Figure 9).

Serious Adverse Events

**Figure 10:**
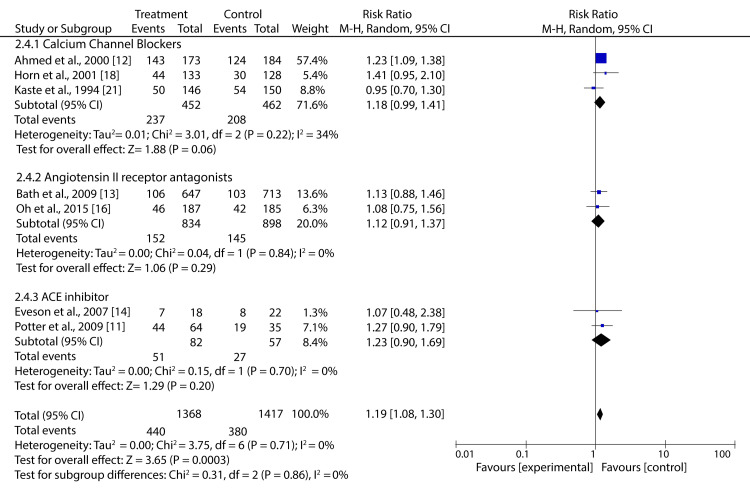
Forest plot showing the effect of HBP control on the risk of recurrent strokes and combined vascular events Ahmed et al., 2000 [12], Horn et al., 2001 [18], Kaste et al., 1994 [21], Bath et al., 2009 [13], Oh et al., 2015 [16], Eveson et al., 2007 [14], Potter et al., 2009 [11] HBP: high blood pressure

Three trials reporting events of recurrent strokes suggested that BP lowering therapy has a similar effect on the risk of stroke recurrence as the control (RR = 1.00; 95% CI [0.54, 1.84]; p = 1.00). Similarly, a meta-analysis of three trials showed that BPR had no significant impact on the risk of combined vascular events (RR=0.99; 95% CI [0.70, 1.41]; p = 0.95) (Figure 10).

Discussion

The decision on whether to lower HBP after AIS remains controversial. The current research results suggest that there is no benefit of BPR on the outcome of death or dependency in AIS patients not treated with reperfusion therapies. Moreover, our analysis has revealed that BPR before undergoing reperfusion therapies significantly reduces the dependency of AIS patients. However, after successful reperfusion treatment, BPR reduction seems to increase the dependency risk.

The findings of this study on the risk of mortality among patients not treated with reperfusion treatment are supported by two previous meta-analyses that found no significant change in short-term (<1 month) and long-term mortality (≥1 month or 3-6 months) among patients receiving BPR medications and the control groups [25,26]. In contrast, the Intravenous Nimodipine West European stroke trial (INWEST) found that patients treated with a high dose of nimodipine and achieving a 20% reduction in DBP had a higher incidence of mortality than the placebo group (OR: 4.336; 95% CI: 1.131-16.619) [12]. However, the outcomes of this trial cannot be relied exclusively on to guide clinical care as it had some methodological concerns. First, the study had a small population, meaning it was at risk of small sample size bias. Second, it included patients who were already receiving antihypertensive medications before randomization. Finally, the study used a follow-up of 21 days to record their results, which might not be sufficient to evaluate the outcomes in AIS patients. This was evident when the authors adjusted the study period to 24 weeks and found no significant difference in the high-dose group. 

Moreover, our analysis has revealed that BPR in patients not treated with reperfusion therapies has no significant effect on short-term and long-term dependency outcomes. This finding is supported by four previous meta-analyses [25-28]; however, the Very Early Nimodipine Use in Stroke trial (VENUS) indicated that BPR in individuals with ischemic stroke marginally increased the likelihood of poor functional outcomes at three months [18]. This elevated risk of poor functional outcomes might be related to using nimodipine, which was associated with deteriorating effects in earlier studies [29]. Moreover, the results of this trial might have been affected by the following methodological concerns. The inclusion criteria for patients in this trial were carried out by inexperienced physicians, which may have led to selection bias. Additionally, this trial did not use the most specific stroke scales to assess dependency because untrained general practitioners cannot evaluate them.

HBP before reperfusion treatments is also a significant cause of concern and is the main reason for withholding reperfusion in patients with AIS. Research has shown that HBP during thrombolytic treatment increases the risk for intracerebral hemorrhage (the risk is four times increased when the SBP is >170 mmHg than when it is between 141 and 150 mmHg) [30,31]. Therefore, BPR before the reperfusion therapies in AIS patients seems to be implied; however, very little is known about the effect of BPR in patients undergoing reperfusion treatment. Our meta-analysis of two trials has shown that lowering BP before reperfusion therapies has a neutral effect on mortality outcomes but significantly reduces the dependency of AIS patients. This finding is heavily weighted by the Third International Stroke Trial (IST-3), which evaluated the effect of BPR in patients undergoing reperfusion treatment using intravenous tissue plasminogen activator (tPA) [17]. According to this trial, BPR during the first 24 hours was significantly associated with the reduced risk of poor functional outcomes at six months (OR: 0.78; 95% CI: 0.065-0.93; p = 0.007). However, the study somehow reported higher incidences of recurrent ischemic strokes in patients with more significant BP decline during the first 24 hours. This finding was theoretically explained by critically low perfusion pressure in some patients due to cerebral arterial autoregulation disruption. Moreover, a more considerable decline in BP was associated with a reduced risk of other adverse events, possibly outweighing the high incidences of recurrent strokes.

Additionally, research has shown that BPR after reperfusion can prevent ischemia-reperfusion lesions [32]; however, it could potentially increase the risk of extensive infarct growth with incomplete or unsuccessful reperfusion [33]. Therefore, investigating the impact of BP management in AIS patients after reperfusion therapies is of great interest. Our meta-analysis of two trials revealed that aggressive BPR after successful reperfusion has a neutral effect on mortality risk but seems to increase the risk of dependency. However, the heterogeneity between the studies was significantly high (I2 = 78%), indicating a wide outcome variation. Therefore, more high-quality randomized trials are required to confirm our results.

The present review also shows that BPR has no remarkable influence on the incidence of recurrent strokes. This finding is supported by two previous meta-analyses [27,28] and one randomized study [34], which revealed that BPR has moderate to no significant impact on the risk of stroke recurrence. Conversely, a subgroup analysis study indicated that after 24 hours, the risk of recurrent strokes was considerably lower in patients receiving antihypertensive medications than in the control group (p = 0.01) [19]. The reduced risk was equivalent to an odds reduction of 25% (95% CI: 8-74). However, the data in that research cannot offer a firm response to guide clinical treatment as it is structured as a subgroup analysis prone to poor statistical power and multiple comparison mistakes [35].

Our review suggests that HBP reduction after AIS has no considerable influence on the occurrences of vascular events. This finding is supported by a prior meta-analysis, which revealed a negligible change in the risk of recurrent vascular events after early BPR (p= 0.54) [27]. Although we integrated the vascular events seen after BPR, it is necessary to separate the influence of BPR on each event. In the subgroup analysis study [13], it was found that patients who underwent BPR had higher occurrences of myocardial infarction (MI) than the control group (3 (0.5%) vs. 1 (0.1%), respectively). However, the difference between these two groups was statistically insignificant. Similarly, Oh et al. showed little change in nonfatal strokes, nonfatal MI, and vascular fatalities between patients taking valsartan and the control subjects (p = 0.771) [16].

When addressing HBP in AIS, it is also crucial to analyze the influence of parameters such as drug class and time to treatment on clinical results. Our subgroup analyses have shown that no individual drug class (i.e., calcium channel blockers (CCB), angiotensin II receptor antagonists (ARA), ACE inhibitors, and beta-blockers (BB)) had a significant effect on mortality or dependency of AIS patients. This finding is consistent with the Cochrane review, which found that ACEI, ARA, and BB had a neutral effect on death and dependency outcomes of patients with acute stroke [26]. Moreover, meta-analyses analyzing the effects of calcium antagonists in AIS patients did not find any significant difference in deaths and dependency between the treatment and control groups [36,37].

Nonetheless, considerable variation among the data compromises the suggestion's reliability. This concern will gain additional support when the results of the two ongoing trials [38,39] examining the same topic reach completion.

Nevertheless, our analysis has revealed that in patients treated <24 hours, ≥24 hours, and ≥72 hours after stroke onset, the risk of mortality and dependency did not differ between the treatment and control groups. However, some of the included studies have provided varying information. The VENUS trial included patients who started treatment six hours after stroke onset and found that the treatment group had significantly poorer functional outcomes than the control group [18]. On the other hand, a subgroup analysis in the CATIS trial indicated that patients treated with antihypertensive at ≥24 hours of stroke onset significantly reduced poor functional outcomes [10].

Limitations

This review has several limitations to be accounted for when interpreting the results. It is essential to note that only scientific articles published in English were included, meaning that other studies relevant to our research were omitted that could have otherwise improved the statistical power of the meta-analysis. Subgroup analysis studies and small retrospective studies were also included in our analyses. Unlike randomized trials, these studies are prone to low statistical power and comparison errors, thus introducing bias to our results. The meta-analysis included the Valsartan efficacy on modest BPR in the AIS (VENTURE) trial [16], which was underpowered as a result of the termination of 70% of its planned sample size, therefore, impacting the expected outcomes on death or dependency and thus influencing the results of our meta-analysis. Finally, it can be noted that high heterogeneity was observed in some of the analyses. However, heterogeneity was expected, given that the antihypertensive agents used in each study varied. Similarly, the fact that each agent was administered at different times after the onset of AIS may have also introduced the high heterogeneity in these outcomes.

## Conclusions

In conclusion, this systematic review and meta-analysis provide valuable insights into BP regulation strategies for AIS patients. For those not undergoing reperfusion therapies, there is no evident reduction in mortality or dependency associated with BPR within the initial 24 hours following stroke onset. Therefore, physicians should exercise caution in administering antihypertensive agents during this early period unless specific comorbid conditions necessitate intervention.

Conversely, our analysis suggests a potential benefit regarding reduced dependency when BPR is initiated before reperfusion therapy for AIS patients scheduled for reperfusion treatment. It is recommended to implement BPR measures in this particular patient subset promptly. However, aggressive BPR targeting an SBP of less than 120 mmHg is not advisable, as it is associated with an increased risk of dependency in AIS patients who have successfully undergone reperfusion treatment. Further research is needed to clarify specific drug classes for BP reduction and the optimal timing of such interventions after AIS onset.

## Appendices

Search strategy

Outcome 1: Blood Pressure Lowering in AIS Patients Not Subjected to Reperfusion Treatment

(“acute ischemic stroke” OR "AIS” OR “Cerebrovascular event” OR “ischemic stroke” OR “brain ischemia” OR “brain infarct*” ) AND (“Blood pressure decrease” OR “blood pressure reduction” OR “blood pressure lowering drugs” OR “antihypertensive*” OR “antihypertensive drug*” OR “beta blockers” OR “calcium channel blockers” OR “ACE inhibitors” OR “Beta Blockers” OR “Angiotensin receptor antagonists” OR “nimodipine” OR “Telmisartan” OR “Candesartan” OR “lisinopril” OR “Valsartan” OR “nitrate” OR “glyceryl trinitrate” OR “GTN”) AND (“Mortality” OR “Fatality*” OR “Death”) AND (“dependent” OR “dependency” OR “neurological outcome” OR “good neurological outcome” OR “poor neurological outcome") AND (“randomized trials” OR “RCT” OR “randomized controlled trial” OR “randomized clinical trial”).

Outcome 2: Blood Pressure Lowering in AIS Patients Not Subjected to Reperfusion Treatment

(“acute ischemic stroke” OR "AIS” OR “Cerebrovascular event” OR “ischemic stroke” OR “brain ischemia” OR “brain infarct*” OR “Intracranial arteriosclerosis”) AND (“Thrombolysis” OR “thrombolytic” OR “fibrinolysis” OR “fibrinolytic*” OR “fibrinolytic agent*” OR “urokinase” OR “anistreplase” OR “streptokinase” OR “tPA” OR “plasminogen activator*” OR “tissue plasminogen activator*” OR “recombinant tissue plasminogen activator*” OR “rtPA” OR “rt-PA” OR “alteplase” OR “reteplase” OR “tenecteplase” OR “recombinant protein*” OR “endovascular therapy” OR “endovascular treatment” OR “endovascular procedure*” OR “endovascular method*” OR “endovascular stenting” OR “endovascular thrombectomy”  OR “mechanical thrombolysis” ) AND (“Blood pressure decrease” OR “blood pressure reduction” OR “blood pressure lowering drugs” OR “antihypertensive*” OR “antihypertensive drug*” OR “beta blockers” OR “calcium channel blockers” OR “ACE inhibitors” OR “Beta Blockers” OR “Angiotensin receptor antagonists” OR “nimodipine” OR “Telmisartan” OR “Candesartan” OR “lisinopril” OR “Valsartan” OR “nitrate” OR “glyceryl trinitrate” OR “GTN”) AND (“Mortality” OR “Fatality*” OR “Death”) AND (“dependent” OR “dependency” OR “neurological outcome” OR “good neurological outcome” OR “poor neurological outcome") AND (“randomized trials” OR “RCT” OR “randomized controlled trial” OR “randomized clinical trial”).

Outcome 3: Effect of HBP Control on the Risk of Recurrent Strokes and Combined Vascular Events

(“acute ischemic stroke” OR "AIS” OR “Cerebrovascular event” OR “ischemic stroke” OR “brain ischemia” OR “brain infarct*” OR “Intracranial arteriosclerosis”) AND (“Blood pressure decrease” OR “blood pressure reduction” OR “blood pressure lowering drugs” OR “antihypertensive*” OR “antihypertensive drug*” OR “beta blockers” OR “calcium channel blockers” OR “ACE inhibitors” OR “Beta Blockers” OR “Angiotensin receptor antagonists” OR “nimodipine” OR “Telmisartan” OR “Candesartan” OR “lisinopril” OR “Valsartan” OR “nitrate” OR “glyceryl trinitrate” OR “GTN”) AND (“recurrent strokes” OR “Recurring strokes” OR “stroke recurrence” OR “vascular events” OR "cardiovascular events” OR “nonfatal strokes” OR “myocardial infarction” OR “vascular death”).
